# Circulating MicroRNAs and Cytokines Associated with Celiac Disease

**DOI:** 10.34172/mejdd.2024.388

**Published:** 2024-07-31

**Authors:** Dargham Hammad, Fadyia Mahdi Muslim Alameedy

**Affiliations:** ^1^Department of Pathological Analysis, Faculty of Science, Kufa University, Najaf, Iraq

**Keywords:** Celiac disease, miR-155, miR-15b, Cytokines

## Abstract

**Background:**

The current research examines the molecular terrain of celiac disease (CD) through microRNA (miRNA) and cytokines as potential new diagnostic and therapeutic markers. Gluten-appropriate immune response is a key feature of an autoimmune clinical entity known as CD that leads to inflammation and degeneration of small intestine mucosa. However, the mechanisms responsible for this remain unclear.

**Methods:**

Quantitative reverse transcription polymerase chain reaction (RT-qPCR ) was carried out on serum samples obtained from patients with CD and control groups to unravel their pathogenesis. Assessing miR-155, miR-15b, interleukin (IL)-2, IL-7, IL-35and IL-37 levels in expression might be useful in diagnosing or treating the disorder.

**Results:**

A significant dysregulation of these molecular players in patients with CD compared with healthy controls has been evidenced by results from this study. For instance, miR-155 was up-regulated, whereas miR-15b was significantly down-regulated in CD, illustrating their roles in immune responses and inflammation-mediated processes. Besides, there was an over-expression of IL-2 and an under-expression of IL-37 in patients with CD, indicating these biomolecules’ role in immuno-dysregulation and inflammatory process underlying CD. In addition, a positive correlation between IL-2 and miRNA 155 expression levels was observed in patients with CD, suggesting that they could be involved together with other cytokines, showing the interplay between immune response pathways and inflammatory cascades during CD pathogenesis.

**Conclusion:**

These molecular signature discoveries might result in new and revolutionary diagnostic modalities and molecular-targeted therapies for CD pathogenesis. When used with the scientific understanding of miRNAs and cytokines associated with CD pathophysiology, it creates a basis for personalized medicine based on the individualized molecular profile of all patients. This will undoubtedly increase the efficacy of CD treatment strategies. In brief, more research on molecular pathways’ workings should be done to harness their potential in CD diagnosis and treatment.

## Introduction

 Celiac disease (CD) is an autoimmune enteropathy that affects the small bowel in genetically exposed people. It is triggered via gluten and has numerous clinical symptoms.^1^ People with the HLA-DQ2 and DQ8 haplotypes have an abnormal immunological response to gluten, which leads to chronic inflammation and injury to the duodenal mucosa.^2^ Following a gluten-free diet (GFD) reverses symptoms and intestinal and serological changes.^3^ The GFD is challenging to follow, may have adverse metabolic effects, and generally lowers the patient’s quality of life.^4^ Although our understanding of CD has expanded significantly in recent years, its etiology is still unknown.

 Recent research has highlighted the significance of microRNAs (miRNAs) in CD pathophysiology.^3^ Small RNA molecules known as miRNAs attach to complementary sequences, particularly messenger RNAs’ non-translatable region 3Ϲ, untranslated regions (UTR).^3^ By influencing the translation of essential transcripts, several miRNAs contribute to immune cells’ differentiation, activation, and functionality.^5^ These have been primarily studied in relation to autoimmune diseases and cancer, but they may also be necessary for CD.^3^

 Dysregulation of miRNAs, such as miR-155 and miR-15b, disrupts immune homeostasis, contributing to chronic inflammation in CD.^6^ MiRNA155 is a multifunctional miRNA that controls hematopoiesis, inflammation, and immunological responses.^7^ Inflammatory cytokines and Toll-like receptor (TLR) ligands promote its production.^7^ MiRNA-155 expression has been altered in autoimmune illnesses such as inflammatory bowel disease,^8^ systemic lupus erythematosus,^9^ and rheumatoid arthritis.^10^ MicroRNA-15b (miR-15b) plays a vital role in autoimmune illnesses due to its impact on immune responses, inflammation, and autoimmunity.^11^ MiR-15b dysregulation has been identified in various autoimmune illnesses, influencing the disease’s activity and development.^12^ MiR-15b affects immune cell function, inflammatory pathways, and tissue homeostasis, making it a possible treatment target for autoimmune disorders.^11^

 Furthermore, past studies have found that miRNA dysregulations in CD have been well studied. Baulina and colleagues reported dysregulation patterns of miR-155 and miR-15b in autoimmune disorders, supporting their possible implication in CD.^13^ In addition, Chamani and others demonstrated changes in the expression profiles of miRNA after being exposed to gluten, which indicated a relationship between miRNA dysregulation and the origin of CD pathogenesis.^14^

 Besides miRNAs, cytokines are small proteins secreted by immune cells that regulate inflammation and responses.^15^ Dysregulated cytokines generate uncontrolled immunological responses, hence a chronic inflammation within the small intestine of patients with CD.^16^ Among cytokines, interleukin-2 (IL-2), interleukin-35 (IL-35), interleukin-37 (IL-37), and interleukin-7 (IL-7) have been chosen for investigation due to their roles in immune regulation and inflammation, and their potential relevance to CD pathophysiology. IL-2 and IL-7 are pro-inflammatory cytokines associated with the pathogenesis of CD.^17,18^ IL-2 controls T cell stimulation and proliferation, contributing to mucosal injury and inflammation in CD patients’ small intestines.^17^ Similarly, IL-7 is required for T cell growth and immunological homeostasis maintenance; alterations in IL-7 signaling may contribute to immune dysregulation and inflammation in CD.^18^

 Furthermore, IL-35 and IL-37 are anti-inflammatory cytokines belonging to the IL-12 and IL-1 families.^19^ Their recruitment reduces inflammation in several autoimmune disorders, including multiple sclerosis, psoriasis, and rheumatoid arthritis.^19^ IL-35/IL-37 is primarily produced by regulatory T cells (Tregs) and regulatory B cells. IL-35 and IL-37 regulate the immune system by inhibiting the nuclear transcription factor kappa-B (NF-κB) and mitogen-activated protein kinase (MAPK) signaling pathways and boosting the proliferation of Tregs and Bregs.^19^

 Furthermore, IL-35 and IL-37 can reduce inflammation by balancing the Th17/Treg ratio. Among the anti-inflammatory cytokines, IL-35 and IL-37 are highly effective in reducing inflammation in the intestinal tract.^19^ The research by Singh and co-workers shows that IL-2 is vital in immune responses, especially in autoimmune diseases like CD.^20^ However, Brown and others discovered that its level has decreased when different autoimmune diseases such as CD came into play, IL-37 implying it could be an inflammation suppressor or an immunoregulatory organizer for inflammation in a similar case.^21^

 The other thing is that studies have shown how genetic variations affect miRNA production or cytokine deregulation among patients with CD. This highlights the contribution of genetic factors to the susceptibility and severity of this disease.^22^ These findings enhance our understanding of molecular mechanisms behind CD and highlight therapeutic potential concerning targeting miRNAs or cytokines during managing such conditions. Therefore, our CD study evaluated the potential of specific molecules, including miRNAs and cytokines, as biomarkers for diagnostic and therapeutic purposes.

 Thus, the present study on CD aimed to investigate the utility of certain molecules, notably miRNAs, as biomarkers for diagnostics and therapies. In particular, we examined miR-155, miR-15b, IL-2, IL-7, IL-35, and IL-37 to understand their roles in CD pathophysiology and determine if they may serve as potential biomarkers. Hence, all these molecules are significantly aberrant in patients with CD compared with healthy controls; hence, they can be used as reliable markers reflecting disease progression and therapy outcomes. This means that molecular markers have a high promise for improving the diagnosis and therapy of CD.

## Materials and Methods

###  Participants in the Study

 We collected blood samples from 50 people with CD and the same number of healthy control subjects who volunteered. These were paired by sex and body mass index (BMI) for blood donation intended for research purposes.

 Matching patients and control groups based on BMI is a popular method in research, especially in domains such as medicine. This strategy aids in managing health-related confounding variables, forming more homogeneous and comparable groups, reducing bias, and ensuring clinical relevance, particularly in research involving gastrointestinal disorders. Overall, matching based on BMI improves the validity and reliability of relevant research findings.

 The sample collection was done between May 20th and September 10th, 2023. In every case, the celiac testing proved positive for tissue transglutaminase (TTG) IgA antibody serologic test at diagnosis time. Furthermore, a gastro-duodenoscopy was performed in all patients with CD and duodenal biopsy specimens taken from the second part of the duodenum. This enabled us to confirm CD diagnosis again and evaluate mucosal damage severity. A blinded pathologist then used Marsh modification^5^ to conduct a histological evaluation of biopsy samples. Further blood Samples were properly labeled and stored in ice boxes before transportation to the laboratory within two hours of collection. In the laboratory, serum samples were stored at -80 °C until further analysis, including detecting IL-2, IL-37, IL-7, IL-35, miR-15b, and miR-155 using reverse transcription-quantitative polymerase chain reaction (RT-qPCR). Samples were collected from participants across various hospitals in Najaf province, including Al-Hakim Hospital, AL-Sadder Teaching Hospital, and Al-Farat Hospital.

###  Diagnosing Human Sample Subjects

 Diagnosing human subjects involved performing qPCR using primers listed in Table 1, obtained from NCBI and designed by us, optimized according to the protocol. This technique aimed to amplify human RNA genes to detect IL-2, IL-37, IL-7, IL-35, miR-15b, and miR-155 using the RNA kit (Cat. Number: E2075, EcoPURE). The PCR mixture was prepared to a final volume of 20 μL, comprising Nuclease-Free H2O (1 μL), 2x Add RT-PCR SYBR Master Mix (10 μL), forward primer (10 µM, 2 μL), reverse primer (10 µM, 2 μL), and RNA template (5 μL). The cDNA synthesis was conducted using RT-PCR SYBR Master cDNA (Cat. Number: 21691, Thermo Scientific RevertAid). The PCR conditions included pre-denaturation at 95 °C for 2 minutes (one cycle), denaturation at 95 °C for 30 seconds, annealing at 57.2 °C for 30 seconds, extension at 72 °C for 50 seconds (for 40 cycles), final extension at 72 °C for 5 minutes (one cycle), and holding at 4 °C.

**Table 1:** Primers used in this study

**Type**		**Gene name sequences**	**Bases**	**Product size**
IL-2	Primer F	GTGAATCCAAGCCCAGAAAA	20 bp	232 bp
	Primer R	GGCATGAACAATGTGGAGAA	20 bp	
IL-37	Primer F	CCCCACCATGAATTTTGTTC	20 bp	184 bp
	Primer R	GGATTTGCTTCCACAAAGGA	20 bp	
IL-7	Primer F	TGGGTGACTCAGCTGTTCTG	20 bp	154 bp
	Primer R	TGAGGTCCTGCTCTGTGATG	20 bp	
IL-35	Primer F	AGGCACAAACTCATCCATCC	20 bp	157 bp
	Primer R	GTCAGAACCCAGCGTTTCAT	20 bp	
SFRP-U6	Primer F	GTTTTGTAGTTTTTGGAGTTAGTGTTGTGT	30 bp	135 bp
	Primer R	CTCAACCTACAATCAAAAACAACACAAACA	30 bp	
MicroRNA 155	RT	GTCGTATCCAGTGCAGGGTCCGAGGTATTCGCACTGGATACGACAACCCC	50bp	240 bp
	Primer F	AACACGCTTAATGCTAATCGTGA	23bp	300 bp
	Primer R	GTCGTATCCAGTGCAGGGT	19bp	320 bp
MicroRNA-15b	RT	GTCGTATCCAGTGCAGGGTCCGAGGTATTCGCACTGGATACGACTGTAAA	50bp	240 bp
	Primer F	AACCTCCTAGCAGCACATCAT	21bp	300 bp
	Primer R	GTCGTATCCAGTGCAGGGT	19bp	320 bp

###  Equation Delta Ct equations to Analyze RT-qPCR Results

 The analysis of RT-qPCR results involved calculating the threshold cycle (Ct) value using the following equations:

*ΔCt Value (Control) (ΔCtC) = Average Ct value of the control group (test gene) - Average Ct value of the control group (housekeeping gene)*

*ΔCt Value (Experimental) (ΔCtE) = Average Ct value of the experimental group (test gene) - Average Ct value of the experimental group (housekeeping gene)*

*Delta Delta Ct value (ΔΔCt) = ΔCtE – ΔCtC*

 These equations allowed for the comparison of gene expression levels between the control and experimental groups, providing insights into the relative expression of the target genes.

###  Statistical Analysis

 GraphPad Prism 9 generated all graphs for statistical data analysis. The Mann–Whitney U test was employed to determine significant differences between samples following treatment. The data from both groups were presented as the median with the interquartile range.

## Results

###  Demographic Comparison in Celiac Disease and Healthy Controls

**Figure 1:**
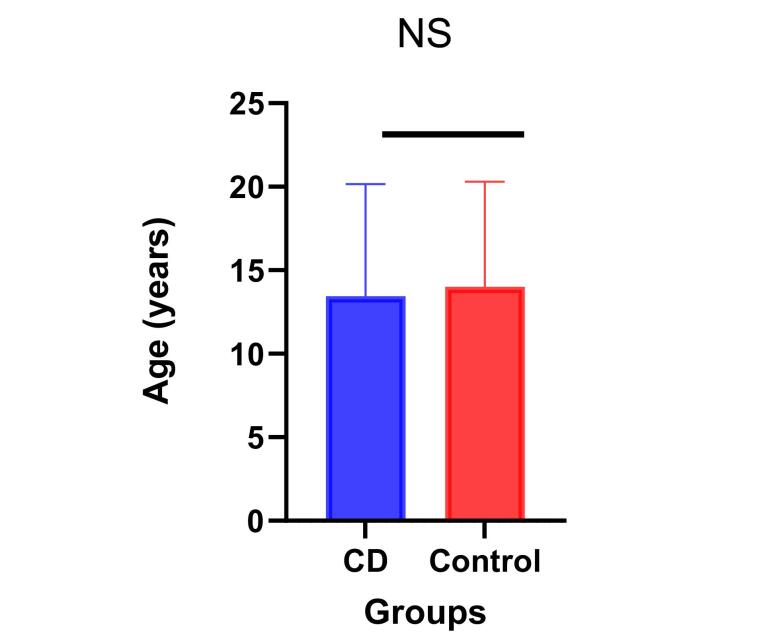
The age (years) of the participants

 The study involved 100 human donors providing whole blood samples. Among them, 50 were diagnosed with CD, all females aged between 2 and 25 years, with an average age (SD) of 13 (6.7) years. Additionally, 50 control blood samples were obtained from female donors aged 3 to 26 years, with an average age (SD) of 14 (6.3) years. Statistical analysis using an unpaired *t*-test revealed that the age differences between the two groups were not significant (*P* > 0.05) (Figure 1).

###  MicroRNA-155 and MicroRNA 15b Expression Level

**Figure 2:**
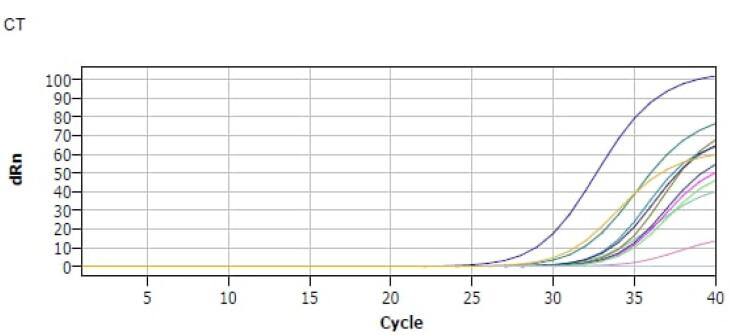
The detection of microRNA-155, microRNA-15b, and U6 snRNA in patients with celiac disease using RT-qPCR

 In the serum samples collected from patients with CD and healthy control subjects, miR-155 and microRNA-15b expression levels were assessed using RT-qPCR. These levels were compared to U6 snRNA, which served as a control for miRNA qPCR. Both microRNA-155, microRNA-15b, and U6 snRNA consistently showed reliable Ct values across all samples from individuals with CD and controls. No replicates with a Ct value greater than 35 were detected in the analysis (Figure 2).

**Figure 3:**
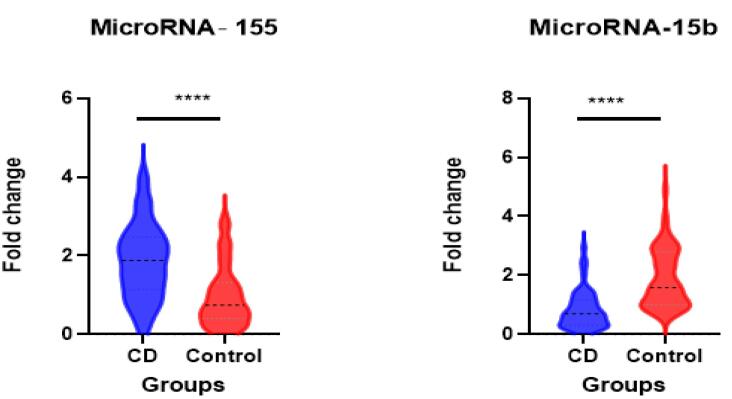
Serum microRNA-155 and microRNA-15b expression levels in patients CD and healthy controls. Total RNA was extracted from the serum of patients with CD (n = 50 and healthy control subjects (n = 50) and was reverse transcribed to cDNA using miRNA-specific primers. The expression levels of serum microRNA-155 and microRNA-15b relative to U6 snRNA were determined by reverse transcription-quantitative polymerase chain reaction using a TaqMan assay. The statistical significance between groups was determined using the Mann-Whitney test, with ****P < 0.0001. Data were presented as the median with an interquartile range for both cohorts. In the graphical representation, the blue violin plot represented individuals with celiac disease, while the red represented healthy controls

 Our study reveals a significant upregulation of miR-155 (*P* < 0.0001) and downregulation of microRNA-15b (*P* < 0.0001) in patients diagnosed with CD compared with healthy controls. Specifically, the median fold change of miR-155 was 1.78 in patients with CD and 0.7 in healthy controls, while the median fold change of microRNA-15b was 0.7 in patients with CD and 1.6 in healthy controls (Figure 3).

###  Cytokine Dysregulation in Celiac Disease

**Figure 4:**
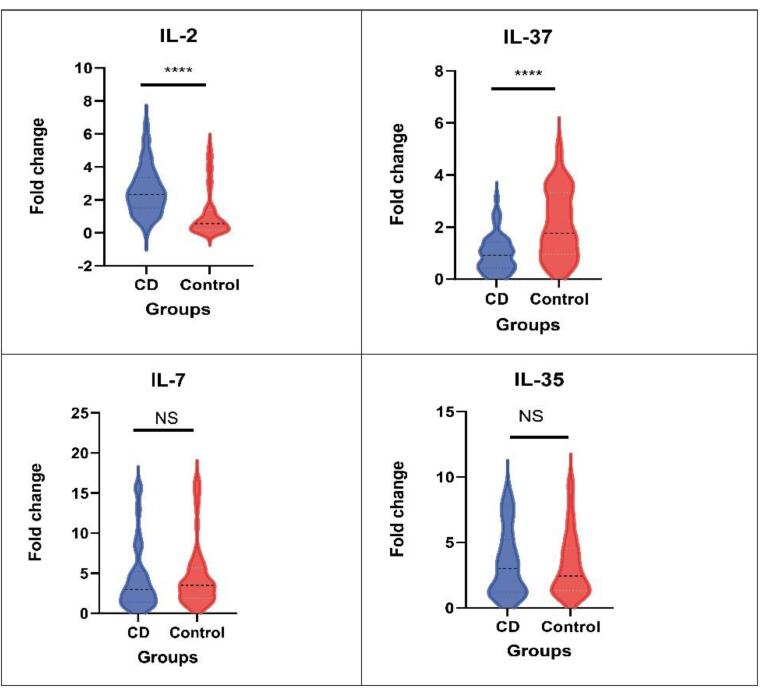
The fold change in gene expression of four cytokines (IL-2, IL-37, IL-7, and IL-35) was detected in 50 patients with celiac disease compared with 50 healthy individuals. Violin plots visually represented each cytokine’s expression. Data were presented as median with interquartile range for both groups. In the graphical representation, patients with celiac disease were represented by the blue violin plot. In contrast, healthy control subjects were represented by the red one, with ****P < 0.0001 indicating significant differences and “NS” indicating no significant difference

 The study investigated the expression levels of various cytokines, including IL-2, IL-37, IL-7, and IL-35, in patients with CD and healthy controls, which were assessed using RT-qPCR. These levels were compared to U6 snRNA, which served as a control for miRNA qPCR. This analysis aimed to discern any disparities in cytokine expression between the two groups, shedding light on cytokine dysregulation associated with CD. The Mann-Whitney test found that patients with CD exhibited a significantly higher fold change in IL-2 expression than healthy controls (median fold change 2.3 vs. 0.56, *P <*0.0001). Conversely, a notably lower fold change in IL-37 expression was observed in patients with CD compared with healthy controls (median fold change 0.9 vs. 1.76, *P <*0.0001). These findings underscore the potential role of IL-2 and IL-37 dysregulation in CD pathogenesis (Figure 4).

###  MicroRNAs’ Correlation with Cytokine Levels in Patients with Celiac Disease

**Figure 5:**
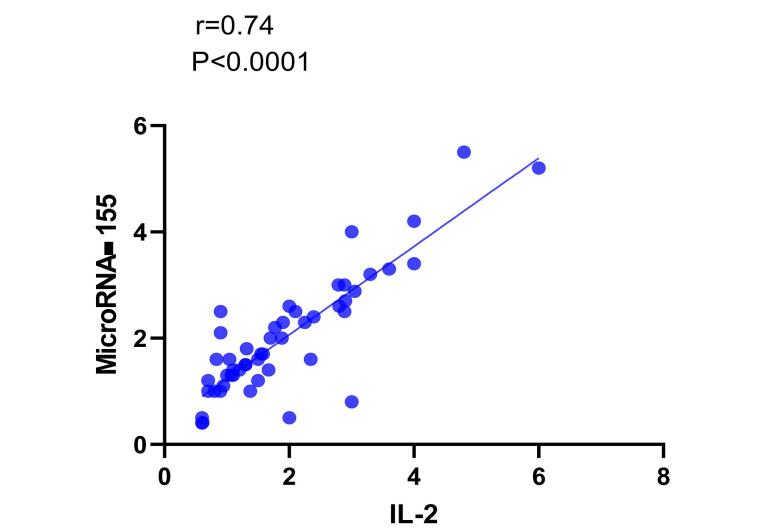
The correlation between miRNA-155 and IL-2 levels in the serum of patients with celiac disease was examined. A scatter plot curve illustrated a strong positive correlation between miRNA-155 and IL-2 (r = 0.74, *P* < 0.0001). Statistical analysis was conducted using the Spearman correlation test, with a statistical significance threshold set at a 95% confidence level (*P* < 0.05). Significant data were those with *P* < 0.05

 Our analysis investigated the correlations between serum levels of microRNA-155 and microRNA-15b with the levels of tested cytokines in patients with CD. The Spearman correlation test revealed a statistically significant positive correlation between microRNA-155 and IL-2 levels (r = 0.74, *P* < 0.0001) (Figure 5).

## Discussion

 Investigating the miRNAs and cytokines expression profiles in CD provides crucial insights into the underlying molecular mechanisms of this complex autoimmune disorder. In our study, we used RT-qPCR to analyze the levels of miR-155, microRNA-15b, and various cytokines, including IL-2, IL-37, IL-7, and IL-35, in serum samples from both patients with CD and healthy controls. The expression levels of the miRNAs and cytokines in patients with CD were dysregulated through our analysis by comparing them with U6 SNRNA as an internal control. These data have unraveled some possible molecular markers and pathways involved in CD pathogenesis.

 This is why the disorganization of miR-155 and miRNA-15b is significant for individuals with CD. To this end, it is essential to grasp such mechanisms to understand more deeply how these miRNAs participate in disease progression. Other works carried out by Liu et al,^11^ and Huang et al^12^ pointed out similar patterns of miR-155 and miRNA-15b dysregulation in autoimmune diseases and inflammatory conditions,^23-31^ which further suggest their possible role in CD pathophysiology.

 Both miR-155 and miRNA-15b play indispensable roles in immune regulatory networks. It closely regulates many aspects of host immunity, like T-cell, B-cell, dendritic cell activation, and differentiation.^32^ Additionally, it modulates the functions of immune cells alongside cytokine production by micro RNA 15b. As a result, malfunctions or disruptions brought about by these small RNAs could disrupt immune homeostasis, leading to chronic inflammation seen in CD.^33,34^ The significance of MiR 155 has been established as an inflammatory response mediator. Its upregulation in patients with CD may exacerbate intestinal inflammation and tissue damage through enhanced immune cell activation and cytokine release.^35^ Conversely, the downregulation of microRNA-15b, known for its anti-inflammatory properties, may further intensify inflammation and tissue injury in CD.^36^

 miR-155 and microRNA-15b maintain epithelial barrier integrity in the gastrointestinal tract. Disruption of the epithelial barrier is a hallmark feature of CD and contributes to increased intestinal permeability and mucosal damage.^34,37^ miR-155 has been shown to regulate tight junction proteins and mucin production, thereby influencing epithelial barrier function.^38,39^ Also, dysregulation of these miRNAs might compromise epithelial barrier integrity in CD, increasing antigen exposure and immune activation.

 Additionally, research on gluten-induced alterations in miR-155 and microRNA-15b expression profiles in intestinal epithelial cells and immune cells has indicated that gluten exposure might trigger CD.^40,41^ Consequently, these modifications of miRNA expression could cause uncontrolled inflammatory responses in patients with CD, leading to chronic inflammation and tissue injury. Moreover, genetic variations may affect miR-155 and microRNA-15b expression levels observed among patients with CD. It is also quite probable that polymorphisms affecting their regulation within a CD or impacting gene target sites for miRNAs are also quite probable. The inability to properly regulate disease severity and susceptibility can be caused by dysregulation of these molecules through changes within their sequence-binding regions due to SNPs affecting them.^42,43^

 Our studies found considerable evidence of immune dysfunction relative to healthy individuals in patients with CD.^20^ Specifically, we noted a significant increase in IL-2 expression; this pro-inflammatory cytokine is known for its high production by activated T cells.^20^ This discovery matches the existing literature, emphasizing the IL-2 role in controlling immune responses, especially concerning autoimmune disorders like CD.^20^ The abnormal expression of IL2 augments the activation of T-cells by gluten antigens, leading to an inflammatory cascade and gut inflammation in small intestines.^44^

 On the other hand, a noticeable decrease in the levels of IL-37 was observed among people with CD.^45^ IL-37, known for its anti-inflammatory attributes and regulatory tasks toward immune responses, is a natural suppressor of inflammation.^46^ The fall in expression of IL-37 is consistent with observations made on other autoimmune conditions where reduced cytokine levels are associated with disordered immunity and progression of diseases.^47^ Low concentrations of this protein will cause uncontrolled inflammation and weakening immunity; as such, a chronic inflammatory environment typical for people with CD will be maintained at a high level.^48^ It is essential to understand how these two pro-inflammatory molecules, IL-2 and IL-37, are misregulated so that their implications can be determined during pathology.^49^ Genetic predispositions, modifications in immune cell subsets, and changes in signaling cascades may likely be some of the causes of the observed cytokine expression alterations.^50^

 This inconsistency disrupts the delicate balance between pro-inflammatory and anti-inflammatory cytokines, hence possibly fueling immune dysregulation and disease progression among patients with CD.^51^

 These findings further emphasize the need to understand the basis of IL-2 and IL-37 dysregulation, an essential step toward developing innovative treatment options.^52^ Restoring immune balance by controlling levels of these two molecules is a potential strategy for reducing inflammation in patients with CD, leading to less damage to the gut lining and a better quality of life.^53^

 Different factors can explain why microRNA-155 and IL-2 levels are correlated in people with CD. To begin with, microRNA-155’s influence on T cell differentiation and activation can result in increased production of IL-2 by activated T cells.^54^ It could also react with signaling, epigenetic regulation of IL-2 gene expression, or other regulatory pathways promoting its secretion,^55^ suppress other cytokines that are important in this disorder,^56^ regulate immune cell subpopulations associated with intolerance to gluten-containing products^57^ and control cytoplasmic events linked to cytokine receptor; such as JAK/STAT3 pathway capable of triggering pSTAT5 phosphorylation-dependent mechanism transducing signal from it into nucleus regulating genes responsible for ILs specific reactions to them.^58^ Therefore, more details about how these mechanisms operate will be necessary for understanding their role in miR155-associated changes in IL-2 and immunological responses toward CD.

## Limitations

 There are important insights to be gained in a study on miRNA and cytokine dysregulation in CD, but the study also has limitations. This study only had female participants, which may mean it overlooked gender-specific differences in CD. Given possible sub-group analyses, this could result in reduced statistical power and representativeness due to the small sample size. Male exclusion thus amounts to selection bias, which does not capture disease characteristics and responses to treatment. Besides, the clinical relevance of the study is limited by its failure to consider heterogeneity in CD and confounding factors such as ethnicity, medication use, and dietary habits. Consequently, these weaknesses need addressing for future research purposes so that we can understand more about CD and better care for patients.

## Conclusion

 This study examines the dysregulation of miRNAs and cytokines in CD. The expression levels of miR-155 were up-regulated. In contrast, miR-15b levels were down-regulated with elevated IL-2 and decreased IL-37 expression levels, indicating an immune imbalance in patients with CD compared with controls. Their involvement in the pathogenesis of CD is suggested by miR-155’s association with IL-2. These findings identify new potential diagnostic and therapeutic targets for CD. Further research is needed to understand these molecular pathways and optimize their clinical applications.
